# Stress hyperglycemia is predictive of clinical outcomes in patients with spontaneous intracerebral hemorrhage

**DOI:** 10.1186/s12883-022-02760-9

**Published:** 2022-06-27

**Authors:** Sijia Li, Yu Wang, Wenjuan Wang, Qian Zhang, Anxin Wang, Xingquan Zhao

**Affiliations:** 1 Department of Neurology, Beijing Tiantan Hospital, Capital Medical University, No.119 South 4th Ring West Road, Fengtai District, Beijing, 100070 China; 2 China National Clinical Research Center for Neurological Diseases, No.119 South 4th Ring West Road, Fengtai District, Beijing, 100070 China; 3 Research Unit of Artificial Intelligence in Cerebrovascular Disease, Chinese Academy of Medical Sciences, No.119 South 4th Ring West Road, Fengtai District, Beijing, 100070 China

**Keywords:** Stress hyperglycemia, Glucose-to-HbA1c ratio, Intracerebral hemorrhage, Prognosis

## Abstract

**Background:**

Stress hyperglycemia is a common condition in patients suffering from critical illness such as spontaneous intracerebral hemorrhage (ICH). Our study aimed to use glucose-to-glycated hemoglobin (HbA1c) ratio to investigate the impact of stress hyperglycemia on clinical outcomes in patients with ICH.

**Methods:**

A sample of eligible 586 patients with spontaneous intracerebral hemorrhage from a multicenter, hospital-based cohort between 2014 and 2016 were recruited in our study. Stress hyperglycemia was evaluated by the index of the glucose-to-HbA1c ratio that was calculated by fasting blood glucose (mmol/L) divided by HbA1c (%). Patients were divided into two groups based on the median of the glucose-to-HbA1c ratio. The main outcomes were poor functional outcomes (modified Rankin Scale score of 3–6) at discharge and 90 days. Multivariable logistic regression and stratified analyses were performed to explore the association of stress hyperglycemia with poor prognosis of ICH.

**Results:**

On multivariable analysis, higher glucose-to-HbA1c ratio (≥1.02) was independently correlated with poor functional outcomes at discharge (adjusted OR = 3.52, 95%CI: 1.98–6.23) and 90 days (adjusted OR = 2.27, 95%CI: 1.38–3.73) after adjusting for potential confounding factors. The correlation between glucose-to-HbA1c ratio and worse functional outcomes still retained in patients with or without diabetes mellitus.

**Conclusions:**

Stress hyperglycemia, calculated by glucose-to-HbA1c ratio, was independently correlated with worse functional outcomes at discharge and 90 days in patients with ICH. Moreover, glucose-to-HbA1c ratio, might not only be used as a simple and readily available index to predict clinical outcomes of ICH but also provide meaningful insight into future analysis to investigate the optimal range of glucose levels among ICH patients and develop tailored glucose-lowering strategies.

**Supplementary Information:**

The online version contains supplementary material available at 10.1186/s12883-022-02760-9.

## Background

Intracerebral hemorrhage remains the second leading cause of stroke [1–3], leading to a higher mortality rate and severe neurological deficits [4, 5]. Considering that there are limited treatment options for ICH patients [4, 6], early detection and management of the risk factors for adverse consequences is extremely essential to optimize outcomes.

Stress hyperglycemia refers to the relative transient condition of elevated blood glucose levels, which is commonly observed in critical illness such as myocardial infarction, ischemic stroke and intracerebral hemorrhage [7–9]. Several previous studies have indicated that stress hyperglycemia was related to elevated risk of death and poor functional outcomes following ICH although these trends have not been described consistently [7, 10–14]. In addition, in most previous studies, stress hyperglycemia was usually defined as absolute hyperglycemia based on the random or fasting blood glucose levels without excluding the effect of chronically background hyperglycemia [7, 8, 12]. Glycated hemoglobin (HbA1c) is a reliable measure of the mean levels of glucose concentration over 2–3 months before the onset of the acute illness [15, 16]. In recent studies, glucose-to-HbA1c ratio, which assessed the degree of the stress hyperglycemia with consideration of the background glucose concentration has gained its popularity and has shown a better predictive value for outcomes of critical illness than absolute hyperglycemia [9, 17]. Moreover, this novel parameter is associated with unfavorable clinical outcomes in patients with ischemic stroke [18–23]. However, the effect of stress hyperglycemia on the poor prognosis of ICH with taking the background glucose levels into account has been rarely discussed in multi-center studies with relatively large sample size and consecutive information of functional outcomes.

Therefore, in our study, we aimed to investigate the association between stress hyperglycemia, which was calculated by glucose-to-HbA1c ratio, and clinical outcomes in patients with ICH.

## Methods

### Study design and population

The study was a prospective, multicenter, observational cohort study, conducted in 13 hospitals in Beijing from 2014 to 2016. In each participating center, clinical data was collected and submitted it online to the coordinating center at Beijing Tiantan Hospital, Capital Medical University for further analysis. The study was performed in accordance with the ethical guidelines from the Helsinki Declaration and was approved by the Institutional Review Board (IRB) of Beijing Tiantan Hospital, Capital Medical University. All the patients or their legal proxies signed the written informed consent.

The inclusion criteria were 1) ICH was diagnosed based on WHO standard and confirmed by CT scan, 2) first-ever acute-onset ICH, 3) age of 18 years or older and 4) arriving at hospital within 72 h after symptom onset.

**Fig. 1:**
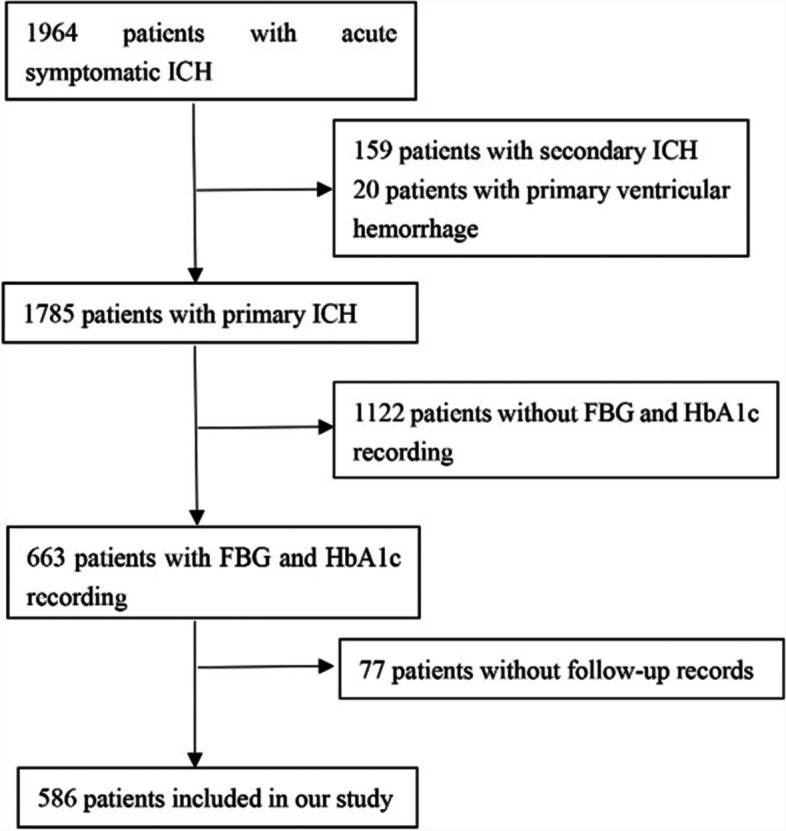
Flow diagram of study patients. ICH: intracerebral hemorrhage; FBG: fasting blood glucose; HbA1c: Glycated hemoglobin

The exclusion criteria were patients complicated with major comorbidities or late-stage diseases. A total of 1964 ICH patients were screened. The additional exclusion criteria of our analysis were as follows: 1) patients with secondary ICH, which attribute to trauma, aneurysms, cerebral venous thrombosis, cerebrovascular malformations, tumor or hemorrhagic transformation of ischemic stroke, 2) patients with primary ventricular hemorrhage, 3) patients without the fasting blood glucose and HbA1c recording and 4) patients without the follow-up information. Finally, 586 patients were included in this study (Fig. 1).

### Baseline information

Baseline information including demographics (age and sex), medical history (hypertension, diabetes mellitus, dyslipidemia and cerebral infarction) and health habits (smoking and alcohol consumption) were documented using standard questionnaires by trained research coordinators. Hypertension was defined as a self-reported history, receiving any antihypertensive treatment or a systolic blood pressure ≥ 140 mmHg, or diastolic blood pressure ≥ 90 mmHg at baseline. Dyslipidemia was noted as a self-reported history, taking lipid-lowering medicine, or a total cholesterol level ≥ 6.22 mmol/L or low-density lipoprotein ≥4.14 mmol/L or triglyceride ≥2.26 mmol/L at baseline. Diabetes mellitus was diagnosed when a patient had a prior history of diabetes or a HbA1c level of ≥6.5% [24]. Smoking was noted when a patient smoked at least one cigarette per day for over a year. Alcohol consumption was documented as an intake of at least 80 g of liquor 1 day for over a year. Prior use of antidiabetic agents and treatment of hyperglycemia during hospitalization were also collected. In this study, anti-hyperglycemic therapy was determined by our stroke physicians based on the patient’s blood glucose levels and general clinical condition and we do not have a standard prescribing routine for blood glucose management during hospitalization.

Stroke severity was assessed using the Glasgow Coma Scale (GCS) and the National Institutes of Health Stroke Scale (NIHSS) by physicians at the timing of arriving at hospital. On the initial CT scan, which was performed within 24 hours after admission, we documented the hematoma volume (ABC/2 method) [25] and location (lobar, basal ganglia, thalamus,,cerebellum brainstem).

### Laboratory examinations and assessment of stress hyperglycemia

Fasting blood glucose was measured the next morning after an overnight fast for at least 8 hours. HbA1c was measured during the first 7 days of acute hospitalization. Other laboratory examinations, including triglyceride (TG), total cholesterol (TC), high-density lipoprotein cholesterol (HDL-C), low-density lipoprotein cholesterol (LDL-C), and high- sensitivity C-reactive protein (Hs-CRP) were also evaluated during admission. Estimated glomerular filtration rate (eGFR) was calculated based on the Chronic Kidney Disease Epidemiology Collaboration creatinine equation with an adjusted coefficient of 1.1 for the Asian population [26].

Stress hyperglycemia was evaluated by the index of the glucose-to-HbA1c ratio that was calculated by fasting blood glucose (mmol/L) divided by HbA1c (%) [17]. The reasons for using fasting blood glucose as numerator instead of admission random blood glucose were its better predictive value on stroke outcomes [27–31] and almost unaffected by food or other sugary infusion [27, 32] as well as its little variation between individuals [27]. The patients were then categorized into 2 groups according to the median of the glucose-to-HbA1c ratio for further comparisons. The index, which takes the baseline blood glucose levels into consideration, quantifies the extent of stress hyperglycemia.

### Follow-up information and clinical outcome

All the patients were followed up by face-to-face interviews at discharge and by telephone interviews at 90-day after ICH onset. Trained research personals, who were blinded to patients’ clinical information, followed the interview protocol to assess the scores of mRS according to the functional status reported by the patients or their relatives or caregivers at each follow-up. An unfavorable clinical outcome was defined as a score of 3 to 6 on mRS.

### Statistics analysis

Categorical variables were expressed as numbers (proportions), while continuous variables were described as means ± standard deviation (SD) or medians (interquartile range, IQR) as appropriate. Continuous variables that were normally distributed were compared using Student t-tests while if were not distributed normally, Wilcoxon rank-sum tests were applied. For categorical variables, chi-squared tests were used to perform comparisons. Multivariate logistic regression model was performed to calculate the odds ratios (OR) and 95% confidence intervals (CI) for the relationship between glucose-to-HbA1c ratio and poor clinical outcomes. Potential confounders with *p*-value<0.2 in comparisons of baseline characteristics grouped by glucose-to-HbA1c ratio were included in multivariate analysis. Confounders that were known to be correlated with poor clinical outcomes after ICH were also entered into our multivariate model. These variables included age, gender, current smoking, alcohol consumption, hypertension, diabetes mellitus, dyslipidemia, history of cerebral infarction, prior antidiabetic agents, systolic blood pressure, GCS score, NIHSS score, location of hematoma, hematoma volume, hsCRP, eGFR, post-stroke treatment of hyperglycemia, surgical treatment and intraventricular extension. Moreover, stratified analysis was conducted according to the diagnosis of diabetes mellitus. Receiver operating characteristic (ROC) curves and area under the curves (AUC) were used to evaluate the predictive power of glucose-to-HbA1c ratio for unfavorable clinical outcomes. All statistical analyses were conducted by SAS software (version 9.4; SAS Institute, Cary, North Carolina, USA). A two-sided *P* < 0.05 was considered to be statistically significant.

## Results

**Fig. 2:**
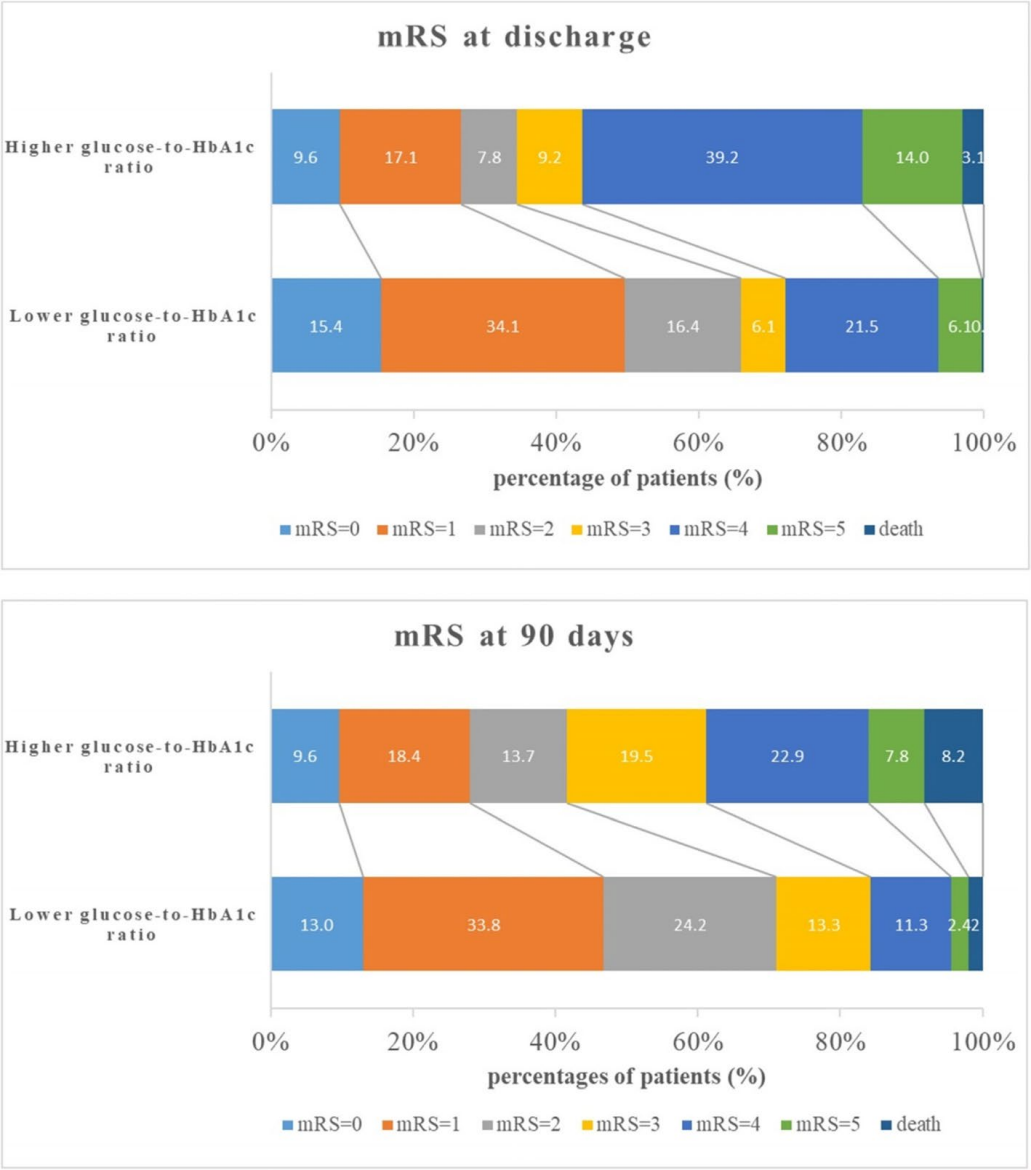
Distribution of scores on mRS at discharge and 90 days in different glucose-to-HbA1c ratio groups. mRS: modified Rankin Scale; HbA1c: Glycated hemoglobin

A total of 586 patients were finally recruited in the present study. The mean age was 58.5 years old, among which 412 (70.3%) patients were male. As the median of the glucose-to-HbA1c ratio was 1.02, patients were classified into a higher glucose-to-HbA1c ratio group and a lower glucose-to- HbA1c ratio group and their baseline characteristics were presented in Table 1. Patients in the higher glucose-to-HbA1c ratio group were significantly older, had higher proportion of diabetes mellitus, and had higher systolic blood pressure, lower GCS score as well as higher NIHSS score. Initial hematoma volume in the higher glucose-to-HbA1c ratio group was larger and was more prone to break into ventricle. Additionally, patients with elevated glucose-to-HbA1c ratio had higher level of FBG and were more likely to receive treatment of hyperglycemia and surgical intervention. For the poor functional outcome, the proportions of disability or death decreased from discharge to 90-day follow-up in both groups, but remained higher in patients with elevated glucose-to-HbA1c ratio (65.5% vs 34.1% at discharge and 58.4% vs 29.0% at 90-day respectively, all *p* < 0.001) (Table 1, Fig. 2). The baseline characteristics between included and excluded participants in the present study were shown in Additional file 2. Excluded patients were younger, less likely to smoke or drink and had lower proportions of hypertension, diabetes mellitus, dyslipidemia, history of cerebral infarction, prior antidiabetic agents and post-stroke treatment of hyperglycemia. We also found that patients in the excluded group had lower GCS scores, higher NIHSS scores, larger hematoma volume, more often intraventricular extension and tend to receive surgical treatment.

**Table 1:** Baseline characteristics of the participants according to median of the glucose-to-HbA1c ratio

	Overall(*n* = 586)	glucose-to-HbA1c ratio		*P* value
		<1.02(*n* = 293)	≥1.02(*n* = 293)	
Male, n (%)	412 (70.3)	209 (71.3)	203 (69.2)	0.59
Age (years)	58.5 ± 13.2	56.9 ± 12.7	60.1 ± 13.5	<0.01
Current smoking, n (%)	207 (35.3)	97 (33.1)	110 (37.5)	0.12
Alcohol consumption, n (%)	252 (43.0)	126 (43.0)	126 (43.0)	0.42
Hypertension, n (%)	560 (95.6)	276 (94.2)	284 (96.9)	0.11
Diabetes mellitus, n (%)	144 (24.6)	58 (19.8)	86 (29.4)	<0.01
Dyslipidemia, n (%)	218 (37.2)	117 (39.9)	101 (34.5)	0.17
History of cerebral infarction, n (%)	96 (16.4)	46 (15.7)	50 (17.1)	0.66
Prior antidiabetic agents, n (%)	75 (12.8)	29 (9.9)	46 (15.7)	0.1
SBP (mmHg)	163.0(150.0–181.0)	160.0 (148.0–177.0)	168.0 (150.0–188.0)	<0.001
DBP (mmHg)	96.0 (83.0–107.0)	95..0 (84.0–105.0)	98.0 (83.0–109.0)	0.21
GCS score	14.0 (12.0–15.0)	15.0 (13.0–15.0)	14.0 (11.0–15.0)	<0.001
NIHSS score	9.0 (3.0–15.0)	7.0 (3.0–13.0)	10.0 (3.0–16.0)	<0.001
Location of hematoma, n (%)				<0.01
lobar	102 (17.4)	49 (16.7)	53 (18.1)	
deep	363 (62.0)	199 (67.9)	164 (56.0)	
infratentorial	50 (8.5)	20 (6.8)	30 (10.2)	
Hematoma volume (ml)	13.0 (5.5–29.4)	10.0 (4.5–21.2)	18.7 (7.4–36.5)	<0.001
intraventricular extension, n (%)	176 (30.0)	59 (20.1)	117 (39.9)	<0.001
eGFR (ml/min)	54.7 (51.1–58.2)	55 .0 (51.6–58.3)	54.3 (50.4–58.0)	0.08
FBG (mmol/l)	5.8 (4.9–7.1)	4.9 (4.4–5.4)	6.8 (6.0–8.2)	<0.001
HbA1c (%)	5.6 (5.3–6.1)	5.6 (5.3–6.1)	5.6 (5.2–6.2)	0.86
hsCRP (mg/l)	8.7 (4.6–8.7)	8.7 (2.8–8.7)	8.7 (8.7–11.1)	<0.001
Post-stroke treatment of hyperglycemia, n (%)	114 (19.5)	41 (14.0)	73 (24.9)	<0.01
Surgery, n (%)	86 (14.7)	14 (4.8)	72 (24.6)	<0.001
Poor functional outcome at discharge, n (%)	292 (49.8)	100 (34.1)	192 (65.5)	<0.001
Poor functional outcome at 90 days, n (%)	256 (43.7)	85 (29.0)	171 (58.4)	<0.001

Continuous variables are expressed as means ± (SD) or medians (IQR)

*SBP* systolic blood pressure, *DBP* diastolic blood pressure, *GCS* Glasgow Coma Scale, *NIHSS* National Institutes of Health Stroke Scale, *eGFR* estimated glomerular filtration rate, *FBG* fasting blood glucose, *HbA1c* Glycated hemoglobin, *hsCRP* high-sensitivity C-reactive protein

On multivariable analysis, higher glucose-to-HbA1c ratio (≥1.02) remained to be independently correlated with poor functional outcomes at discharge (OR 3.52 95%CI 1.98–6.23) and 90 days (OR 2.27 95%CI 1.38–3.73) after adjusting for potential confounding factors (Table 2).

**Table 2:** Crude and adjusted odd ratios of outcomes at discharge and 90-day

	glucose-to-HbA1c ratio	
	<1.02(*n* = 293)	≥1.02(*n* = 293)
Poor outcome at discharge		
Events, n (%)	100 (34.1)	192 (65.5)
Crude OR (95% CI)	1.00 (reference)	3.67 (2.61–5.16)
Adjusted^a^ OR (95% CI)	1.00 (reference)	3.52 (1.98–6.23)
Poor outcome at 90 days		
Events, n (%)	85 (29.0)	171 (58.4)
Crude OR (95% CI)	1.00 (reference)	3.43 (2.43–4.83)
Adjusted^a^ OR (95% CI)	1.00 (reference)	2.27 (1.38–3.73)

*HbA1c* Glycated hemoglobin, *OR* odd ratio, *CI* confidence interval

^a^Adjusted for age, gender, current smoking, alcohol consumption, hypertension, diabetes mellitus, dyslipidemia, history of cerebral infarction, prior antidiabetic agents, systolic blood pressure, GCS score, NIHSS score, location of hematoma, hematoma volume, hsCRP, eGFR, post-stroke treatment of hyperglycemia, surgery and intraventricular extension

Subgroup analysis revealed that diabetes mellitus had no interaction effect on the relationship between glucose-to-HbA1c ratio and unfavorable clinical outcomes in patients with ICH (p for interaction>0.05), although some OR values were significant in some subgroups (Table 3).

**Table 3:** Multivariate-adjusted^a^ OR and 95% CI for poor outcome according to median of the glucose-to-HbA1c ratio

Outcome	Subgroup	glucose-to-HbA1c ratio		*P* for interaction
		<1.02	≥1.02	
Poor outcome at discharge	With diabetes	1.00 (reference)	1.96 (0.29–13.21)	0.85
	Without diabetes	1.00 (reference)	4.16 (2.15–8.04)	
Poor outcome at 90 days	With diabetes	1.00 (reference)	3.82 (0.91–16.05)	0.18
	Without diabetes	1.00 (reference)	2.07 (1.16–3.68)	

*HbA1c* Glycated hemoglobin, *OR* odd ratio, *CI* confidence interval

^a^Adjusted for age, gender, current smoking, alcohol consumption, hypertension, diabetes mellitus, dyslipidemia, history of cerebral infarction, prior antidiabetic agents, systolic blood pressure, GCS score, NIHSS score, location of hematoma, hematoma volume, hsCRP, eGFR, post-stroke treatment of hyperglycemia, surgery and intraventricular extension

**Fig. 3:**
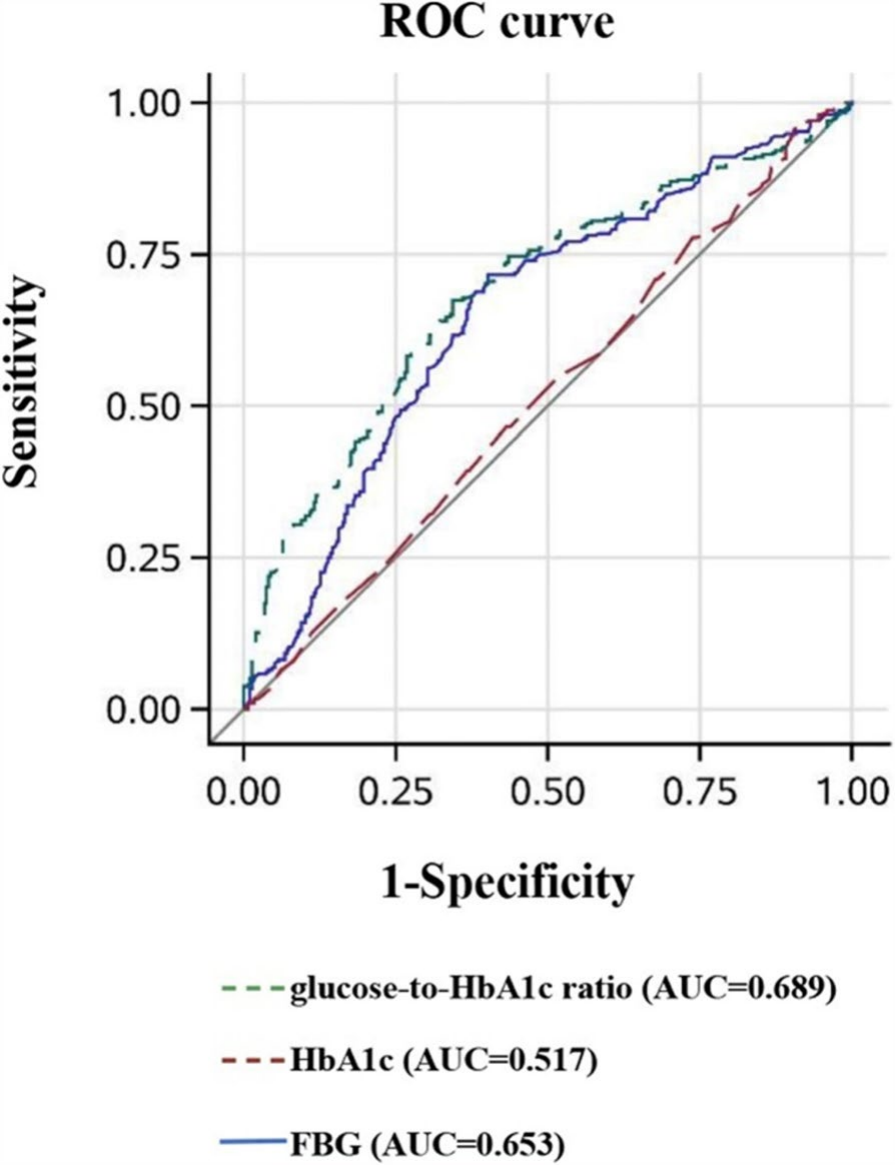
The ROC analysis of glucose-to-HbA1c ratio, FBG and HbA1c levels for predicting poor outcomes at discharge. ROC: receiver operating characteristic; AUC: area under the curve; FBG: fasting blood glucose; HbA1c: Glycated hemoglobin

**Fig. 4:**
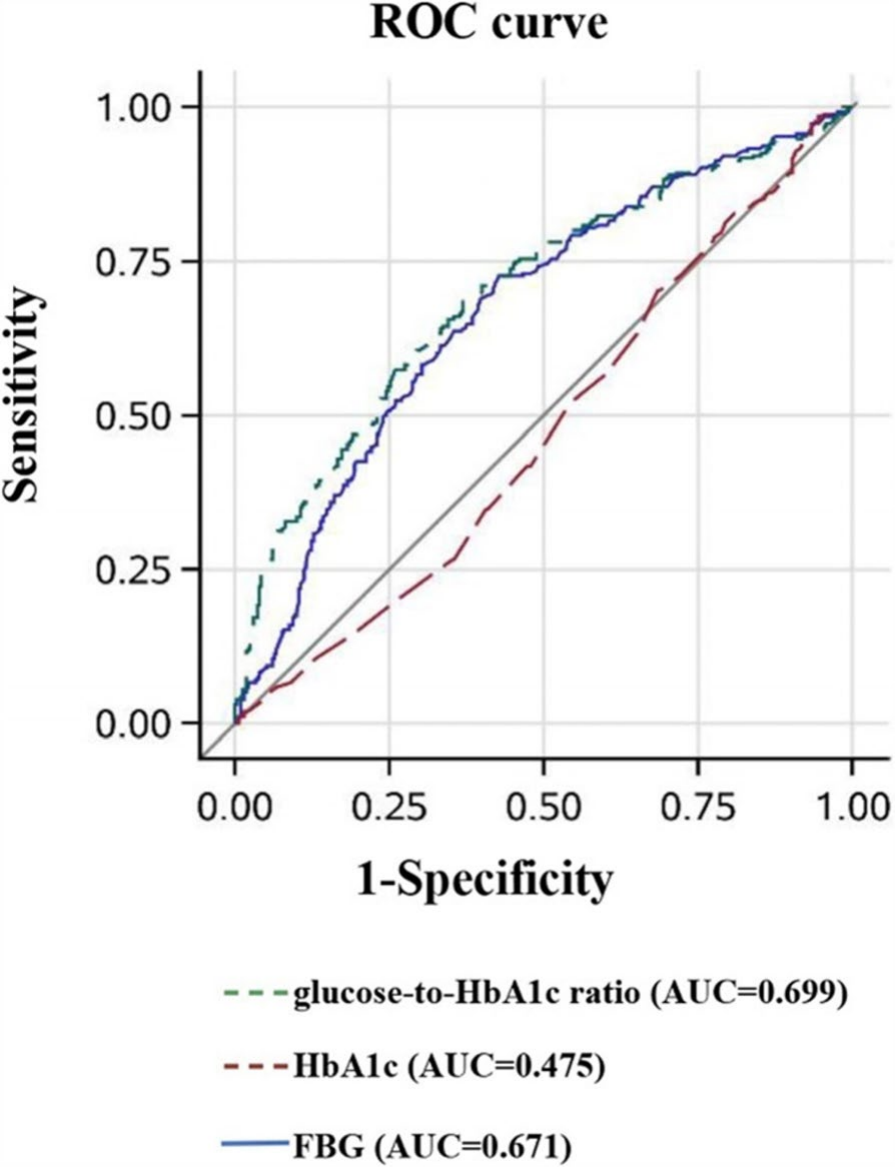
The ROC analysis of glucose-to-HbA1c ratio, FBG and HbA1c levels for predicting poor outcomes at 90-day. ROC: receiver operating characteristic; AUC: area under the curve; FBG: fasting blood glucose; HbA1c: Glycated hemoglobin

Furthermore, the ROC curve analysis demonstrated that glucose-to-HbA1c ratio produced greater AUC values (0.689, 95% CI: 0.646–0.732) than did FBG (AUROC: 0.653, 95% CI: 0.608–0.698, *P* = 0.01) and HbA1c (AUROC: 0.517, 95% CI: 0.471–0.564, *P<*0.0001) for predicting poor clinical outcomes at discharge in pairwise comparison (Fig. 3). In terms of poor clinical outcomes at 90 days, similar results were observed for the superior predictive value of glucose-to-HbA1c ratio (0.699, 95% CI: 0.656–0.742) than did FBG (AUROC: 0.671, 95% CI: 0.627–0.715, *P* = 0.04) and HbA1c (AUROC: 0.475, 95% CI: 0.429–0.522, *P<*0.0001) (Fig. 4).

## Discussion

In this prospective, observational cohort study conducted in Beijing, we discovered that stress hyperglycemia represented by glucose-to-HbA1c ratio was independently associated with poor clinical outcomes at discharge and 90 days in patients with ICH. The correlation still retained irrespective of diabetes mellitus. In addition, our data also implicated that glucose-to-HbA1c ratio had greater discriminative power compared to FBG and HbA1c in predicting poor functional outcomes of ICH.

There were conflicting results regarding the relationship between stress hyperglycemia and poor clinical outcomes in patients with ICH. Prior studies have found that stress hyperglycemia was correlated with increased risk of early mortality and long-term death in patients with ICH [14, 33–35]. Other studies also demonstrated that hyperglycemia was a surrogate marker to predict poor functional outcome at discharge and 90 days after ICH [12, 36–38]. However, some research did not indicate a significant association between hyperglycemia and poor prognosis in patients with ICH [10, 11]. A meta-analysis also shown that stress hyperglycemia did not appear to be independently related to short-term mortality after hemorrhagic stroke [7]. The discrepancies between different studies may be contribute to the study population, confounding factors adjusted in multivariate analysis as well as the definition of stress hyperglycemia. In most of the studies, stress hyperglycemia was usually measured as the absolute hyperglycemia without considering the previous diabetes status or deterioration of pre-illness blood glucose control with preexisting diabetes mellitus [7, 8]. Moreover, given that the absolute hyperglycemia failed to take the background hyperglycemia into account, it was difficult to differentiate stress hyperglycemia from newly diagnosed or previously unknown diabetes mellitus. In contrast, glucose-to-HbA1c ratio, a novel index we used to calculate the degree of stress hyperglycemia, represents a reliable assessment of relatively elevating blood glucose levels, hence can eliminate the influence of background hyperglycemia. Based on recent studies, relative measurements adjusting for prior glycemic status, such as glucose-to-HbA1c ratio or admission glucose level divided by estimated average blood glucose derived from HbA1c, could facilitate better prediction for poor outcomes of critical illness than absolute elevated glucose concentration [9, 17]. Also, HbA1c levels, reflecting the previous glycemic control over 2–3 months [15–17], are characterized by lower biological variability and remain stable even at the acute phase of diseases [39, 40], thus can accurately quantify the degree of stress hyperglycemia. Moreover, relative measurements to asses stress hyperglycemia were considered to be useful indicators of adverse cardiovascular and cerebrovascular events after percutaneous coronary intervention [41], and also elevated risk of poor functional outcomes, all-cause death and hemorrhagic transformation after acute ischemic stroke [18, 19, 42]. In the current study, we further elaborated that stress hyperglycemia, represented by glucose-to-HbA1c ratio could better predict the unfavorable functional outcomes in patents with ICH.

Although the mechanisms underlying the correlation between stress hyperglycemia and poor functional outcomes in patients with ICH are incompletely understood, several possible explanations may account for our findings. First of all, stress hyperglycemia reflects the relative rapid increase of blood glucose and is secondary to a status of neuroendocrine derangements and inflammatory response that occur after an acute major diseases like stroke [18, 19, 42]. Neuroendocrine derangements and inflammatory response are characterized by glycogenolysis, excessive output of glucose from hepatic storage and increasing insulin resistance [9, 21, 43]. Therefore, we can speculate that stress hyperglycemia may be an indicator of the extent of severe stroke, which contribute to poor prognosis after ICH occurs. However, it is interesting to notice that stress hyperglycemia remained significantly associated with poor functional outcomes in patients with ICH even after adjusting the NIHSS score and hematoma volume, further indicating that stress hyperglycemia represents not merely the severity of stroke or not just simply an epiphenomenon of stress response. Second, stress hyperglycemia might exert a direct toxic damage to the brain tissue by exacerbating the accumulation of lactate and intracellular acidosis [20, 23, 36]. And intracellular acidosis could further aggravate free radical formation and lipid peroxidation, which in turn may accelerate the process of nerve injuries [7, 23, 44]. Third, stress hyperglycemia could cause the acute rising of free calcium in the cytoplasm that results in calcium influx into mitochondria and then interferes with the process of ATP generation, leading to cytotoxic edema and neuronal apoptosis [45–47]. Fourth, the downregulation of aquaporin-4 expression in the brain induced by stress hyperglycemia can exacerbate the blood brain barrier destruction, which promotes severe vasogenic brain edema [12, 48], eventually leading to worse functional outcomes after ICH. In addition, stress hyperglycemia is responsible for aggravating hematoma enlargement via plasma kallikrein [13, 34, 49], further resulting in unfavorable outcomes when ICH occurs.

Previous studies found that stress hyperglycemia contributed to worse functional outcomes after ICH particularly in patients without diabetes mellitus [12, 35, 38]. This could be possibly explained by the better tolerance to hyperglycemia in diabetic patients because of the cellular adaptation to chronically exposure to high blood glucose levels [21, 22]. However, in our subgroup analysis, stress hyperglycemia, measured by glucose-to-HbA1c ratio did not have the effect on poor functional outcomes after ICH stratified by diabetes status. This discrepancy might due to the different definition of stress hyperglycemia. Our study, using the glucose-to-HbA1c ratio to calculate stress hyperglycemia rather than the absolute rising of blood glucose levels, have taken the background hyperglycemia into account, further implicating those diabetic patients could also have stress hyperglycemia.

There are several limitations in our study that should be considered. First of all, the cause-and-effect relationship between stress hyperglycemia and clinical outcomes was not verified due to the nature of the observational study. Therefore, further studies should be conducted to establish the causal link. Second, we enrolled patients within 72 hours after symptom onset and patients without the FBG or HbA1c recording or loss to follow-up were excluded. Considering that the mortality rate of ICH within the first 48 h may be as high as 14.9% [50] and the excluded patients had worse neurological status,lower levels of consciousness and larger hematoma volume, thus the present study rule out the most severe patients. Furthermore, participants included in our study were all come from Beijing, China. Therefore, selection bias might exist and our findings may not be generalizable to patients with more serious clinical conditions and wider racial diversity. Thirdly, the extent of hematoma expansion at the early stage was not measured in our study cohort, which may potentially influence the relationship between stress hyperglycemia and ICH outcomes. At the same time, moderate and severe persistent hyperglycemia could have an impact on disability or death after ICH [51]. However, the temporal profile of stress hyperglycemia assessed by glucose-to-HbA1c ratio was not documented, and the influence of severity and duration of stress hyperglycemia on prognosis of ICH was not explored further. Hence, the impact of the dynamic change of glucose-to-HbA1c ratio on ICH outcomes requires further investigation in our future analysis. Finally, some parameters, such as the glycemic variability and the glycemic control were not documented in our study, which could have the residual confounding effects on the functional outcomes.

## Conclusions

In conclusion, our study indicated that stress hyperglycemia, calculated by glucose-to-HbA1c ratio was independently associated with worse functional outcomes at discharge and 90 days in patients with ICH. Moreover, glucose-to-HbA1c ratio, might not only be used as a simple and readily available index to predict clinical outcomes of ICH but also provide meaningful insight into future analysis to investigate the optimal range of glucose levels among ICH patients and develop tailored glucose-lowering strategies.

## Supplementary Information

**Additional file 1.** Included the source of data generated or analyzed during this study. It is an excel file encompassing 2 separated tables. The first table of the excel file including the specific value of each variable while the second table of the excel file including the meaning of each variable.

**Additional file 2.** Included the baseline characteristics and their comparisons between included and excluded participants.

## Data Availability

All data generated or analyzed during this study are included in Additional file 1.

## Abbreviations

ICH: Intracerebral hemorrhage
HbA1c: Glycated hemoglobin
FBG: Fasting blood glucose
GCS: Glasgow Coma Scale
NIHSS: National Institutes of Health Stroke Scale
TG: Triglyceride
TC: Total cholesterol
HDL-C: High-density lipoprotein cholesterol
LDL-C: Low-density lipoprotein cholesterol
Hs-CRP: High- sensitivity C-reactive protein
eGFR: Estimated glomerular filtration rate
mRS: Modified Rankin Scale score
SD: Standard deviation
IQR: Interquartile range
OR: Odds ratio
CI: Confidence interval
ROC: Receiver operating characteristic
AUC: Area under the curves
