# Supplementary material for: Stress hyperglycemia is predictive of clinical outcomes in patients with spontaneous intracerebral hemorrhage
Source: BMC Neurol. 2022 Jun 27;22:236. doi: 10.1186/s12883-022-02760-9 (PMC9235136; doi:10.1186/s12883-022-02760-9)
Supplement: Supplementary file 2 — Additional file 2. Included the baseline characteristics and their comparisons between included and excluded participants. [file 12883_2022_2760_MOESM2_ESM.pdf]

Additional File 2. Baseline characteristics and their comparisons between included and excluded participants

	Included N=586	Excluded N=1378	P value
Male, n(%)	412 (70.3)	915 (66.4)	0.09
Age(years)	58.5±13.2	56.1±14.8	0.0017
Current smoking, n(%)	207 (35.3)	421 (30.6)	< 0.001
Alcohol consumption, n(%)	252 (43.0)	464 (33.7)	< 0.001
Hypertension, n(%)	560 (95.6)	1260 (91.4)	0.0013
Diabetes mellitus, n(%)	144 (24.6)	205 (14.9)	< 0.001
Dyslipidemia, n(%)	218 (37.2)	308 (22.4)	< 0.001
History of cerebral infarction, n(%)	96 (16.4)	171 (12.4)	0.0187
Prior antidiabetic agents, n(%)	75 (12.8)	97 (7.0)	< 0.001
SBP (mmHg)	163.0 (150.0-181.0)	164.5 (147.0-188.0)	0.23
DBP (mmHg)	96.0 (83.0-107.0)	95.0 (82.0-110.0)	0.83
GCS score	14.0 (12.0-15.0)	13.0 (7.0-15.0)	< 0.001
NIHSS score	9.0 (3.0-15.0)	12.0 (4.0-24.0)	< 0.001
Location of hematoma, n(%)			< 0.001
lobar	102 (17.4)	199 (14.4)	
deep	363 (62.0)	631 (45.8)	
infratentorial	50 (8.5)	145 (10.5)	
Hematoma volume (ml)	13.0 (5.5-29.4)	17.8 (6.8-43.1)	< 0.001
intraventricular extension, n (%)	176 (30.0)	479 (34.8)	< 0.001
eGFR (ml/min)	54.7 (51.1-58.2)	55.2 (50.6-59.2)	0.21
hsCRP (mg/l)	8.7 (4.6-8.7)	8.7 (8.7-8.7)	0.04
Post-stoke treatment of hyperglycemia, n(%)	114 (19.5)	154 (11.1)	< 0.001
Surgery, n(%)	86 (14.7)	280 (20.3)	0.0033

Continuous variables are expressed as means ± (SD) or medians (IQR).

SBP: systolic blood pressure; DBP: diastolic blood pressure; GCS: Glasgow Coma Scale; NIHSS: National Institutes of Health Stroke Scale; eGFR: estimated glomerular filtration rate; hsCRP: high-sensitivity C-reactive protein
